# Dietary Selenium Regulates microRNAs in Metabolic Disease: Recent Progress

**DOI:** 10.3390/nu13051527

**Published:** 2021-05-01

**Authors:** Xin Huang, Yu-Lan Dong, Tong Li, Wei Xiong, Xu Zhang, Peng-Jie Wang, Jia-Qiang Huang

**Affiliations:** 1 Beijing Advanced Innovation Center for Food Nutrition and Human Health, Department of Nutrition and Health, China Agricultural University, Beijing 100083, China; 15501852343@163.com (X.H.); ylbcdong@163.com (Y.-L.D.); leetong0606@163.com (T.L.); xiongwei910702@126.com (W.X.); xuzhang91@foxmail.com (X.Z.); wpj1019@cau.edu.cn (P.-J.W.); 2 Key Laboratory of Precision Nutrition and Food Quality, Department of Nutrition and Health, Ministry of Education, China Agricultural University, Beijing 100083, China; 3 College of Veterinary Medicine, China Agricultural University, Beijing 100083, China

**Keywords:** diseases, metabolism, microRNA, selenium, selenoproteins

## Abstract

Selenium (Se) is an essential element for the maintenance of a healthy physiological state. However, due to environmental and dietary factors and the narrow safety range of Se, diseases caused by Se deficiency or excess have gained considerable traction in recent years. In particular, links have been identified between low Se status, cognitive decline, immune disorders, and increased mortality, whereas excess Se increases metabolic risk. Considerable evidence has suggested microRNAs (miRNAs) regulate interactions between the environment (including the diet) and genes, and play important roles in several diseases, including cancer. MiRNAs target messenger RNAs to induce changes in proteins including selenoprotein expression, ultimately generating disease. While a plethora of data exists on the epigenetic regulation of other dietary factors, nutrient Se epigenetics and especially miRNA regulated mechanisms remain unclear. Thus, this review mainly focuses on Se metabolism, pathogenic mechanisms, and miRNAs as key regulatory factors in Se-related diseases. Finally, we attempt to clarify the regulatory mechanisms underpinning Se, miRNAs, selenoproteins, and Se-related diseases.

## 1. Introduction

Selenium (Se) is a metalloid element that fulfills important physiological functions within the necessary dose, but human health is also vulnerable to selenium deficiency or selenium excess [1]. Daily food can meet people’s demand for selenium, comprising a balanced selection of meat and plant products [2]. In the natural environment, rock and soil composition are believed to determine Se distribution characteristics [3]. Populations in the United States, Mexico, Colombia, India, and Iceland easily attain their recommended daily Se dose. However, populations in Northern Europe, Australia, New Zealand, and China have poor Se-containing soils, potentially leading to Se deficiencies [4].

Recently, microRNAs (miRNAs) and their role in Se-related inflammation and diseases have attracted considerable attention [5,6,7,8]. MiRNAs are non-coding endogenous single-stranded RNA molecules that consist of 20–23 nucleotides [9]. They play central roles in cell differentiation, proliferation, and survival by binding to complementary target messenger RNA (mRNA), leading to mRNA translation, inhibition, and/or degradation [10]. Therefore, they can be regarded as key gene expression regulators that can control physiological and pathological processes, including the development of cancer [9]. Studies have confirmed miRNA dysregulation is causal in many cancers [11,12,13,14,15], with miRNAs acting as tumor suppressors or oncogenes. Similarly, miRNA mimics and molecules targeting miRNAs have shown promise in preclinical studies [12].

Keshan disease (KD) and Kashin–Beck disease (KBD) are related to the Se deficiency, but the actual mechanisms that are behind these diseases are still not precisely understood [16]. Microarray and proteomics analysis revealed the genes and pathways that may be involved in these diseases. Nineteen Se- and three zinc-associated proteins were identified among 105 differentially-expressed proteins. The proteins involved in hypoxia-inducible factor-1α and apoptosis pathways may play significant roles in the pathogenesis of KD [17]. There are numerous functional pathways and cellular systems associated with the differentially expressed genes and proteins; the TCA Cycle II (Eukaryotic) pathway and NADP repair pathway may also participate in the pathogenesis of KD [18]. Thirty-four nutrients associated with differentially expressed genes and ten significant pathways have been identified for juvenile KBD, which are mainly related to metabolism, cell apoptosis, extracellular matrix, and other functions which consist of pathological changes of KBD [19]. One hundred and twenty-four miRNAs had lower expression levels in the subchondral bone sampled from KBD patients showed by miRNA array profiling [20]. These differential genes or proteins may become new targets for studying microRNAs in the two diseases.

In this review, we discussed recent studies related to miRNA changes in cardiovascular diseases, cancer, and other metabolic diseases caused by Se deficiency or excess. The prospect of miRNA as a potential target for selenium-related diseases is also pointed out in the article.

## 2. Selenium Uptake and Metabolism

**Figure 1:**
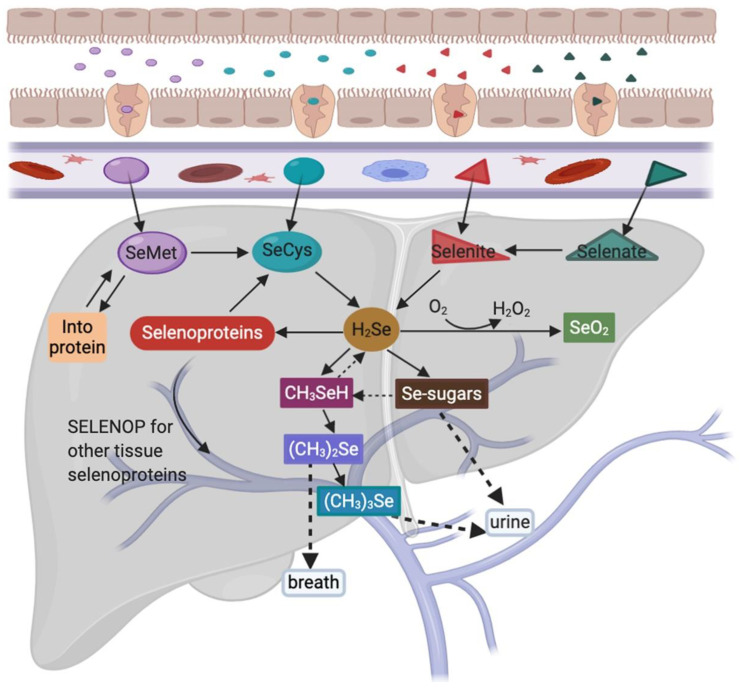
The metabolism of Se dietary forms. SeMet, (selenomethionine); SeCys, (selenocysteine); H_2_Se, (dihydrogen selenide); SELENOP, (selenoprotein P); CH_3_SeH, (methyl selenol); (CH_3_)_2_Se, (dimethyl selenide); (CH_3_)_3_Se^+^, (trimethyl selenonium); SeO_2_, (selenium dioxide).

Se levels in a given food product does not mean an organism will derive its correct Se quota—instead, this depends on the bioavailability, bioaccessibility, and/or bioactivity of a given Se compound [2]. Human dietary Se forms mainly include organic and inorganic Se. These forms are typically absorbed without any regulatory processes and have a high bioavailability in the body [2,21]. Se is primarily absorbed in the duodenum and caecum after active transport via a sodium pump, but this process is different depending on the chemical form [22]. After intestinal absorption, different Se forms enter the bloodstream and are transported into the liver via the portal vein, where they are metabolized, transported, and distributed to different tissues [23] (Figure 1, adapted from [24,25,26,27] and drawn with https://app.biorender.com/ access on 1 March 2021). In normal diets, tissue Se concentrations in the body range from the highest to the lowest in the following organs: Kidney, liver, spleen, pancreas, heart, brain, lung, bone, and skeletal muscle [28].

SeMet accounts for 90% of total Se in plants. Some SeMet is randomly incorporated into proteins at methionine positions [29], whereas other SeMet quantities are metabolized to selenocysteine (SeCys) via methionine cycle and transsulfuration pathways in the liver [26]. Furthermore, Se-methylselenocysteine and γ-glutamyl-Se-methylselenocysteine (believed to exert anticancer effects) are also found in plants, such as garlic, onions, and broccoli, and they are metabolized to methyl selenol [25,30]. SeCys occurs at much lower levels than SeMet in plants. When SeCys is absorbed, free SeCys does not appear to generate concentrations for efficient attachment to cysteine transfer RNA (tRNA). But once incorporated into proteins, SeCys predisposes these proteins to degradation processes [31]. SeCys is the main Se source in animal products, however, highly reactive free SeCys is maintained at very low concentrations in tissues [32]. Inorganic Se is the main form of Se supplementation as it promotes selenoprotein biosynthesis [26]. Selenate must be reduced to selenite before further metabolism. Then, interactions with the tripeptide and glutathione ensure this selenite is reduced to selenide (H_2_Se), which is a central gateway molecule for Se utilization and excretion [25]. Furthermore, all seleno-compounds must be metabolized to selenide for incorporation into selenoproteins [29]. After initial Se metabolism, H_2_Se is converted to selenophosphate, which is used to convert phosphoseryl-tRNA^[Ser]Sec^ to SeCys-tRNA^[Ser]Sec^. Then the SeCys-tRNA^[Ser]Sec^ reads the UGA codon and integrates SeCys into the amino acid sequence to form a selenoprotein [33]. Selenoprotein P, which is mainly produced in the liver, transports Se from the liver to extrahepatic tissues and organs, where it is metabolized to prevent oxidative damage [26]. Methyl selenol is demethylated to H_2_Se in the equilibrium reaction, where it and its precursors (SeMet and CH_3_SeCys) may be used as Se sources for selenoprotein synthesis [34]. The oxidation of excess H_2_Se leads to superoxides and other active oxygen species, often with toxic effects [35]. Se excess detoxification occurs via sequential methylation into dimethyl selenide, and is excreted via the breath, whereas Se-sugars and trimethyl selenonium are excreted in the urine [27]. Although all Se forms are excreted from the body at some stage, only Se-sugars are bioavailable. It is not only an excretion metabolite of Se, but also may transport selenium from liver cells to other cells in the body [36].

## 3. Selenium Related Pathogenic Mechanisms and Diseases

In general, when Se plasma levels are less than 85 µg/L, Se deficiency becomes evident in the body [37]. Se deficiency is caused by poor Se dietary intake, and may be induced or aggravated by nutritional, chemical, and infectious stresses. Several Se deficiency animal diseases are related to the co-existing vitamin E deficiencies [38]. Se deficiency causes heart disease (e.g., cardiomyopathy, arrhythmias), infertility, neuronal or neuromuscular diseases, and increased susceptibility to cancer, infection, and heavy metal toxicity [39,40,41,42,43]. The maximum harmless Se concentration is less than 400 μg per day in adults [44]. Excess dietary Se causes adverse effects (selenosis), including acute food poisoning symptoms, such as vomiting, nausea, and diarrhea, as well as chronic toxicity manifested by the hair and nail brittleness and loss, gastrointestinal disturbances, infertility, and nervous system abnormalities [45]. In addition, when Se is excessive, the toxicity of inorganic Se is much lower than that of organic selenomethionine (SeMet) [38].

Both Se excess and deficiency lead to Se-related disease. A recent epidemiological analysis showed that taking 300 µg/d selenium for 5 consecutive years increased all-cause mortality after 10 years in countries with moderately low selenium levels [46]. Dietary Se functions mainly depend on the selenoprotein form that exerts biological effects in the body. Almost all tissues are affected by changes in Se status or selenoprotein expression [47,48,49]. For instance, embryonic lethality caused by trsp gene deletion, which encodes Sec-tRNA for translation, also reflects the importance of selenoproteins to the body [50]. Currently, 25 genes in the human genome have been identified as encoding selenoproteins, with most exhibiting antioxidant activities [51]. Other specific processes include the biosynthesis of deoxyribonucleoside triphosphates for DNA, the reduction of oxidized proteins and membranes, redox regulation of transcription factors, apoptosis regulation, immunomodulation, thyroid hormone regulation, Se transport and storage, protein folding, and the degradation of misfolded proteins in the endoplasmic reticulum [27]. Therefore, it appears the physiological and pathological changes or diseases caused by Se deficiency are primarily mediated by a selenoprotein imbalance [28,52,53,54,55]. Correspondingly, excess Se generates toxicity via several mechanisms [56]: (1) CH_3_Se− formation, which either enters a redox cycle and generates superoxide and oxidative stress, or generates free radicals that bind to and inhibit key enzymes and proteins, (2) SeCys excess, which inhibits Se methylation metabolism, results in hydrogen selenide (intermediate metabolite) accumulation eventually leading to hepatotoxicity and other Se-related adverse effects, (3) excess Se analogs of sulfur-containing enzymes and structural proteins also play roles in avian teratogenesis. Equally, aquatic organisms exposed to high Se doses are at risk of organ damage and genomic mutations, which potentially pose a threat to human food chains [57,58].

## 4. Current Progress in Nutrient Regulation of miRNAs

### 4.1. miRNAs

MiRNAs are derived from intergenic or intragenic (exon and intron) genomic regions [59]. They are usually transcribed by RNA polymerase II from miRNA genes, first forming a ‘primary miRNA transcript’ (pri-miRNA). This transcript is cleaved by a microprocessor complex, comprising the double-stranded RNase III enzyme, DROSHA, and its essential cofactor, the DiGeorge syndrome critical region 8 (DGCR8) protein, generating a short sequence, the ‘miRNA precursor’ (pre-miRNA), which displays a hairpin-like secondary structure [60]. The pre-miRNA is exported to the cytoplasm and processed by DICER, a ribonuclease III enzyme that produces the mature miRNA for final incorporation into an RNA-induced silencing complex (RISC) [61]. Under most conditions, mature RISC represses gene expression post-transcriptionally by binding to 3′ untranslated regions of specific mRNAs, and mediates degradation, destabilization, or translational inhibition, based on target sequence complementarity [62]. MiRNAs are abundant in all cells, are found in extracellular body fluids (e.g., serum, plasma, saliva, and urine), and are implicated in several pathological conditions [63,64]. During some biological processes, miRNAs regulate protein levels of key regulatory factors, or serve as switches to govern gene expression [65]. Post-transcriptional regulation of miRNA can improve the compliance, accuracy, and sensitivity of gene expression regulation [66]. Due to its small molecular size, each miRNA potentially targets hundreds of mRNA molecules [67]. Similarly, each mRNA may be targeted by multiple miRNAs to form complexes and multifaceted regulatory networks [68]. Moreover, miRNAs also regulate DNA methylation and histone modification [69]. It was previously reported that >60% of human coding genes are regulated by miRNAs [70], and >2800 mature miRNA sequences are described in the miRBase 22 repository (http://www.mirbase.org/ access on 5 February 2021). In addition, miRNAs are not only endogenously synthesized, but may be derived from the diet (e.g., milk and plants) [71].

### 4.2. Regulation Mechanisms of Vitamins and Minerals on miRNAs

Abnormal miRNA expression is driven by genetic and epigenetic factors, which are implicated in cancer occurrence and development, and this may be reversed by a variety of dietary components [72]. Considerable evidence has indicated that dietary factors modify miRNA expression and mRNA targets during cancer, including apoptosis, cell cycle regulation, differentiation, inflammation, angiogenesis and metastasis, and stress response pathways [59], however, this topic is outside the remit of our review. Vitamins and mineral nutrients induce miRNA expression by activating transcription factors/response elements, thereby changing gene expression by inducing mRNA degradation or inhibiting translation [73]. Furthermore, vitamins and minerals alter the function of classical epigenetic mechanisms, including DNA methyltransferases (DNMT) and histone-modifying enzymes (e.g., histone deacetylases (HDAC) and histone acetyltransferases), such enzyme regulation regulates gene expression, including miRNAs [73]. Minerals, such as magnesium ions, are located in the small RNA binding domain of the argonaute protein. RISC is a magnesium-dependent protein; therefore, magnesium ions facilitate the binding of miRNA and the argonaute protein, and also help to cleave miRNA targets and regulate argonaute stability [74]. In contrast, DGCR8 forms a highly stable and active complex with heme, therefore when heme is reduced to a ferrous state, the pri-miRNA processing abilities of the DGCR8 complex disappear [75]. In addition, a recent study observed that aluminum sulfate up-regulated miR-125b and miR-146a expression via NF-κB-dependent mechanisms suggesting these miRNAs were involved in astrocyte proliferation and inflammation [76]. It was recently proposed that the environment, including dietary factors, may induce epigenetic changes via three possible mechanisms: (1) Activation/inhibition of chromatin machinery, (2) activation of nuclear receptors by ligands, (3) membrane receptor signal transduction cascades [77], and (4) the involvement of key epigenetic regulatory enzymes including DNMT, DNA desorption methylase, histone acetylase, and HDAC [78]. MiRNAs are also regulated by DNA methylation status in cells—up to 33% of dysregulated miRNA loci exhibit consistent DNA methylation and H3K9 acetylation patterns [79].

### 4.3. Role of Mammalian Target of Rapamycin (mTOR) in Nutrient Regulation of miRNAs

mTOR is central to the nutrient-sensing signaling network [80]. Its activity is regulated by multiple nutrients, such as amino acids and glucose, influencing muscle cell proliferation, differentiation, autophagy, and metabolism [81]. Following cellular nutrient depletion, in particular leucine starvation, mTORC1 becomes inhibited, thereby inhibiting translation initiation and elongation [82]. Studies have reported that miR-1 expression was regulated by mTOR, mainly via protein stability generated by the myogenic transcription factor, MyoD 18, which was located in the upstream enhancer of multiple myogenic miRNAs [83]. Similarly, miR-133 and miR-206 expression patterns were similarly regulated by MyoD 20 [84]. However, the exact mechanism by which mTORC1 regulates the stability of the myogenic transcription factors remains unclear. In addition, mTOR activation also significantly down-regulated miRNA biogenesis by up-regulating Mdm2, which is an important E3 ligase for the ubiquitination of the miRNA processing enzyme, DROSHA 21 [85]. Also, nutrient starvation can induce autophagy by inhibiting mTORC1, to provide an important substrate source for extracellular energy production [86]. Therefore, the nutrient-mTOR-miRNA pathway appears to rely on the typical nutrient-sensing mTORC1 pathway to regulate autophagy [87]. Importantly, the specific regulatory mechanisms underpinning downstream mRNA-mediated alterations have not been fully elucidated.

### 4.4. miRNAs Mediated by Se Status Are Implicated in Disease Development and Progression

In this section, recent developments on miRNA involvement in Se-related diseases are described, including miRNA changes when Se is in deficient, moderate, or excessive status, regulatory miRNA targets, and miRNA roles in Se antioxidant damage or Se-related diseases (Table 1).

Se supplementation reportedly changed miRNA profiles in the intestinal cell line, Caco-2, differentially down-regulating 12 miRNAs under nutrient deficiency conditions [77]. The miRNAs most affected were miR-185, miR-625, miR-203, and miR-429, whereas pathway analyses identified arachidonic acid metabolism, glutathione metabolism, oxidative stress, and mitochondrial respiration as Se-sensitive pathways [88]. In a Se deficiency rat model, five miRNAs from harvested heart tissue (miR-374, miR-16, miR-199a-5p, miR-195 and miR-30e were up-regulated > 5-fold in the Se nutrient deficiency group, when compared with the Se-supplemented group, whereas three miRNAs were down-regulated (miR-3571, miR-675, and miR-450a [78]. Up-regulated miRNAs were involved in signal transduction, cell differentiation, and stress responses, suggesting roles in cardiac function and regulation [89]. A Se pro-longevity mechanism study reported that several miRNAs were altered in response to dietary Se in the mouse liver [3]. Expression levels of 38 miRNAs were altered by Se deficiency compared with Se sufficiency, and the study showed that selenoprotein regulation by miRNAs was not a direct effect [3]. The role of glutathione peroxidase regulation and related miRNAs has also been reported [90,91]. In an intervention study in elderly males given Se and coenzyme Q10 supplements for four years, significant expression differences were observed in >100 miRNAs, with up to 4-fold differences in combined Se and coenzyme Q10 supplementation experiments [92]. Such changes may contribute to underlying clinical mechanisms. Early reports indicated that cardiovascular mortality was reduced, cardiac function improved, and inflammation and oxidative stress indications decreased after Se intervention [92]. Se decreased inflammation by increasing miR-146a expression, decreasing mmu-miR-155, TLR2/6, NF-κB, and MAPK signaling pathway expression in mammary tissue from infected animals, and mammary epithelial cells [93,94]. Although these studies investigated miRNA-mediated Se deficiency or Se antioxidant damage, data for miRNAs implicated in Se excess are limited. When Se is in excess, it potentially increases the risk of metabolic syndrome [95,96].

Apart from the aforementioned transcription factors being implicated in miRNA regulation by Se, it remains unclear how Se precisely regulates miRNAs. Thus, similar to mechanisms involved in nutrition-gene interactions, it is reasonable to speculate that selenium regulates the expression of miRNAs by potentially affecting the epigenetic regulation mechanisms, including DNA methylation and histone modification. Se supplementation may modify global DNA methylation and specific gene regions, possibly via DNMT inhibition [97]. Additionally, dietary Se deficiency may decrease DNA methylation by enhancing trans-sulfonation pathways [98]. Se also alters histone modification via HDAC inhibition of the Se metabolism products, seleno-α-keto acids [97]. Taken together, the current evidence indicates that different DNA hypomethylation mechanisms occur at different Se levels, including (1) the redirection of homocysteine towards transsulfuration pathways and glutathione synthesis during Se deficiency, (2) excess Se competes with S-adenosylmethionine to use the methyl group required for selenium metabolism—consequently, S-adenosylmethionine levels are reduced for DNMT and methylation processes are similarly inhibited and (3) Se affects specific tumor suppressor gene methylation mechanisms, possibly in a sex-dependent manner. Importantly cancer phenotypes are often characterized by the altered methylation of selenoprotein-encoding genes, mainly glutathione peroxidase 3 [99].

**Table 1:** MiRNAs are regulated by selenium (deficiency, moderate, and excess).

Se Status	Micro RNA	Target	Observed Effect	Note	Reference
Se deficiency	*↑miR-181a-5P*	↓SBP2	↓GPX1, GPX4, and SELENOS levels	In C28/I2 human juvenile chondrocytes and DA rats	[100]
	*↑Gga-let-7f-3p*	↓SELENOK	↑Oxidative stress, ERS, and apoptosis	In chicken myoblasts and muscle	[101]
	*↑miR-200a-5p*	↓*TXNRD2, TXNRD3, SELENON, SELENOT, SELENOF* and *SELENOP*	↑Glucose metabolism disorder, cardiomyocyte hypertrophy	In chicken cardiomyocytes	[102]
		↓RNF11	↑Oxidative stress and myocardial necroptosis	In chicken cardiac tissue and cardiomyocytes	[103]
	*↑miR-138-5p*	↓SELENOM	↑Apoptosis, oxidative stress, mitochondrial fission	In chicken chondrocytes	[104]
	*↑miR-544a*	↓SELENOK	Interferes with SELENOK translation	In HepG2 and HuH-7 human hepatocarcinoma cells	[105]
	*↑miR-196-5p*	↓NFκBIA (IκB-α)	↑LPS-induced oxidative stress and inflammation, respiratory mucosal immune dysfunction	In chicken trachea	[106]
	*↑miR-193b-3p*	↓MAML1	↑Hepatocyte apoptosis	In the liver tissues and primary hepatocytes from broilers	[107]
	*↑miR-33-3p*	↓ADAM10	↑Cell cycle arrest and apoptosis	In vivo and in vitro in the chicken kidney	[108]
		↓E4F1	↑Oxidative stress, ERS, and apoptosis	In vein endothelial cells from broilers	[109]
	*↑miR-328*	↓ATP2A2	↑Intracellular Ca^2+^ and cell apoptosis	In H9c2 rat cardiac myoblasts	[110]
	*↑miR-215-5p*	↓CTCF	↑Mitochondrial biosynthesis imbalance, defects in myocardial development	In heart tissue and primary cardiomyocytes from chickens	[111]
		↓PI3K/AKT/TOR	↑ROS, Myocardial autophagy	In cardiomyocytes of chicken	[112]
	*↑miR-1594*	↓TNNT2	↑Ca^2+^	In heart and primary cardiomyocytes from chickens	[113]
	*↑miR-2954*	↓PI3K	↑Autophagy and apoptosis	In heart and primary cardiomyocytes from chickens	[114]
	*↑miR-16-5p*	↓PI3K/AKT	↑Necroptosis	In tracheal tissues and tracheal epithelial cells of chicken	[115]
	*↑miR-128-1-5p*	↓CADM1	↑Tight junction structural damage and cell cycle arrested	In vein tissues and vein endothelial cells from broilers	[116]
	*↑ miR-374, miR-16, miR-199a-5p, miR-195 and miR-30e* *↓ miR-3571, miR-675a and miR-450a*	↑ Wnt/β--catenin	↑ Cardiac dysfunction	In rat heart	[89]
	*↓miR--185*	*↑GPX2, SEPHS2*	↑Altered expression of 12 miRNA and 50 genes	In Caco-2 human intestinal cells	[88]
	*↓miR-29a-3p*	↑TNFR1	Altered expression of selenoprotein genes, ↑necrotic cells	In the pig brain and IPEC-J2 pig intestinal epithelial cells	[117]
	*↓miR-155*	↑TNFRSF1B	↑Oxidative stress-induced apoptosis	In splenic cells and spleen of broilers	[118]
	*↓miR-146a*	↑MAPKs	↑ROS-induced inflammation	In the head kidney of carp	[119]
	*↓miR-7*	↓SELENOP	Both are potential biomarkers of HCC	In HCC patients and HepG2 human hepatocarcinoma cells	[120]
Se moderate	*↑miR-146a*	↓TLR2, TLR6, NF-κB and MAPK	↓*S. aureus*-infected mastitis	In mammary tissues and mammary epithelial cells from mouse	[93]
	*↑miR-125a and miR-125b*	↓Bak and caspase-3	↓Cd-induced apoptosis	In LLC-PK1 porcine renal epithelial cells	[121]
	*↑ miR-29b-3p, miR-30e-5p and miR-19a-3p* *↓ miR-199a-5p, miR-130a-3p and miR-191-5p*	——	↓ Risk of heart failure	In healthy elderly males	[92]
	*↓mmu-miR-155*	↓TNF-α, IL-1β, IL-10, TLR2, NF-κB and MAPKs	↓ *S. aureus*-infected mastitis	In mammary tissues and mammary epithelial cells from mouse	[94]
	*↓miR-224*	↑*ID1*	↓Pb-induced oxidative damage and restoring thyroid hormone disequilibrium	In thyroid tissues of male rats	[122]
	*↓miR-16-5p*	↑PiK3R1 and IGF1R	↓Pb-induced neutrophil apoptosis	From chicken peripheral blood	[123]
	*↓miR-216a*	↑PI3K/AKT	↓Cd-triggered necrosis and apoptosis	In the splenic lymphocytes of common carp	[124]
Se excess	*↑miR-122-5p*	↑BMI, SBP and DBP	↑Risk of MetS	In male adults	[95]
	*↑miR-454-3p and miR-584-5p* *↓miR-375*	A link between Se intake, vitamin D metabolism, and calcium homeostasis	↑miR-375 as a potential biomarker of MetS	In obese women	[96]

miRNAs, genes, or proteins down-regulated/inhibited (↓) or up-regulated/activated (↑). SBP2, SECIS binding protein 2; SELENOK, selenoprotein K; TXNRD2, thioredoxin reductase 2; TXNRD3, thioredoxin reductase 3; SELENON, selenoprotein N; SELENOT, selenoprotein T; SELENOF, selenoprotein F; SELENOP, selenoprotein P; RNF 11, ring finger protein 11; SELENOM, selenoprotein M; NFκBIA (IκB-α), IkappaB-alpha; MAML1, mastermind-like protein 1; ADAM 10, adisintegrin and metalloprotease domain 10; E4F1, E4F transcription factor 1; ATP2A2, sarcoplasmic/endoplasmic reticulum calcium ATPase 2; CTCF, CCCTC-binding factor; TNNT2, Troponin T Type 2; CADM1, cell adhesion molecule 1; GPX2, glutathione peroxidase 2; SEPHS2, selenophosphatesynthase 2; TNFR1, TNF receptor superfamily member 1A; TNFRSF1B, TNF receptor superfamily member 1B; TLR2, toll-like receptor 2; TLR6, toll-like receptor 6; ID1, Inhibitor of DNA binding 1; PIK3R1, phosphoinositide-3-kinase regulatory subunit 1; IGF1R, type 1 insulin-like growth factor receptor; BMI, body mass index; SBP, systolic pressure, and DBP, diastolic pressure.

## 5. Conclusions

These data indicate that miRNAs can interfere with many proteins, including selenoproteins, promoting Se-related diseases. Some miRNAs have shown clear associations with cardiomyopathy pathology, inflammation, and apoptosis. Actually, the effects of Se on epigenetic mechanisms, especially miRNAs, are poorly described and represent an interesting field of study.

As miRNAs are involved in the transcriptional regulation of genes, thus, acting on the maintenance of the functionality of numerous physiological processes. Se benefits may be mediated through its function as a component of small Se-containing metabolites or via its role in selenoprotein activity/regulation [125]. To clarify the mechanism of microRNA regulating Se-related diseases, we should focus not only on the influence of Se status on miRNAs and the regulation of miRNAs on selenoproteins and other key proteins, but also on the effect of miRNAs on Se status in target tissues. It would be interesting to see whether miRNAs interfering with Se incorporation (e.g., targeting SCLY, TRSP) would have a profound effect on disease.

## Abbreviations

GPX1	glutathione peroxidase-1
GPX3	glutathione peroxidase-3
GPX4	glutathione peroxidase-4
HCC	hepatocellular carcinoma
miRNA	microRNA
Se	selenium
SeMet	selenomethionine
SeCys	selenocysteine
H_2_Se	selenide
HSePO_3_^2−^	selenophosphate
SELENOP	selenoprotein P
oncomiRs	miRNAs acting as tumour suppressors or oncogenes
dNTPs	deoxyribonucleoside triphosphates
pri-miRNA	primary miRNA transcript
DGCR8	DiGeorge syndrome critical region 8
pre-miRNA	miRNA precursor
mRNA	messenger RNA
DNMT	DNA methyltransferase
HATs	histone acetylase
HDAC	histone deacetylase
mTOR	Mammalian target of rapamycin
